# Microbial Diversity of Anaerobic-Fermented Coffee and Potential for Inhibiting Ochratoxin-Produced *Aspergillus niger*

**DOI:** 10.3390/foods12152967

**Published:** 2023-08-06

**Authors:** Bao-Hong Lee, Cheng-Hao Huang, Tsung-Yu Liu, Jung-Shiang Liou, Chih-Yao Hou, Wei-Hsuan Hsu

**Affiliations:** 1 Department of Horticulture, National Chiayi University, Chiayi 600355, Taiwan; bhlee@mail.ncyu.edu.tw (B.-H.L.); doodle29068@gmail.com (C.-H.H.); 2 Department of Food Safety/Hygiene and Risk Management, College of Medicine, National Cheng Kung University, Tainan 701401, Taiwan; tino890108@gmail.com (T.-Y.L.); zx860910@gmail.com (J.-S.L.); 3 Department of Seafood Science, National Kaohsiung University of Science and Technology, Kaohsiung 811213, Taiwan; chihyaohou@gmail.com

**Keywords:** coffee fermentation, anaerobic, temperature, microbial diversity, microorganisms

## Abstract

Coffee flavor considerably depends on the fermentation process, with contributing factors including fermentation temperature, oxygen concentration, and microbial diversity. Efficient controlling of the fermentation can improve the quality of coffee beverages. Therefore, several studies on coffee fermentation processes have been conducted in various regions. The objective of this study was to assess the microbial diversity of coffee beans undergoing anaerobic fermentation at various temperatures (4 °C or 37 °C) and fermentation durations (12 h or 36 h) using full-length 16S rRNA sequencing. This analysis aimed to evaluate the inhibitory effects of the fermented metabolites against ochratoxin-producing *Aspergillus niger*. From our results, *Acetobacter* was identified as the dominant microbial community at higher fermentation temperatures, whereas *Leuconostoc* and *Gluconobacter* were the dominant genera at lower temperatures. However, at lower temperatures, changes in microbial communities were relatively slow. This study expands our knowledge of the microbial diversity involved in the anaerobic fermentation of coffee beans in Taiwan. The findings of this study can be used in future research to cultivate microorganisms linked to the quality and improve the quality of coffee beverages through fermentation.

## 1. Introduction

Coffee, cocoa, tea, and wine are globally popular beverages [1,2]. Their consumption is mostly driven by people’s preferences; therefore, the sensory characteristics of the beverages, such as aroma, flavor, and texture, determine their quality. Before they can be sold, these beverages must go through many processing steps [3,4]. From harvesting to roasting, coffee undergoes various processes, including fermentation, which is crucial for the quality of the final product [5].

The main purpose of coffee fermentation is to separate the mucilage layer attached to the parchment. Various conventional methods, including the dry process, that can physically remove the hull after sun drying are known [6]; however, in areas with limited sunlight, extended drying durations may promote fungal contamination of coffee. Therefore, moist techniques that remove the mucilage layer by microbial breakdown have been developed in places with copious water supplies. During microbial metabolism in the mucilage layer, the metabolic products of the microbes serve as precursors and flavor compounds in coffee, resulting in a coffee with floral and fruity [7,8]. Honey process, a hybrid of dry and wet processes, has been developed in some regions. The honey production process combines the benefits of both dry and wet processes [9]. Coffee beverages produced using traditional processes tend to have classic and simple flavors. In the pursuit of more diverse and special coffee flavors, several innovative processes, such as animal gut fermentation and anaerobic fermentation have emerged [10,11]. Várady et al. (2022) summarized the recent mainstream specialty coffee fermentation processes, including dry, wet, honey, self-induced anaerobic, and carbonic maceration fermentation [12]. With the advancement of food science, there is a deeper understanding of the strains and fermentation processes; the flavors of traditional types of processes have become more stable, but new types of processes can provide a wide range of distinct flavors. Fermentation develops a specific type of aroma and flavor; hence, the type of microorganisms used for fermentation is important and affects the final sensory attributes of the coffee product.

Coffee researchers worldwide have noticed that the flavor of coffee can vary depending on the area or farm, even within the same growing region and fermentation process. They studied the microbial composition during coffee fermentation. They found that different regions have different microbial diversities and that the richness and evenness of various bacteria affect the flavor characteristics of coffee. The composition of various bacteria also causes coffee to lean towards a fruity or nutty flavor profile [8,13,14]. Controlling the fermentation conditions to promote the growth of dominant microorganisms can reduce the growth of potentially harmful fungi and enhance the development of the main flavor characteristics of coffee [15,16].

As the link between microorganisms and flavor compounds of coffee is being increasingly understood, the stability and improvement of coffee flavor and quality are gradually being realized [17]. Environmental conditions such as sugars, fermentation temperature, time, and oxygen concentration all have an impact on fermentation [11]. Researchers have used the oxygen-consuming properties of microorganisms in self-induced anaerobic fermentation. This anaerobic fermentation method can effectively improve the quality of coffee produced on farms with poor facilities. Anaerobic fermentation not only enhances coffee quality but also creates a perceptible sensory profile [18]. Another study reported the use of carbonic maceration fermentation in winemaking. In this method, plant fruits are placed in a sealed environment filled with CO_2_ to achieve absolute anaerobic fermentation, causing the fruit to shift from aerobic respiration to anaerobic fermentation. This method leads to better floral and fruit scents in wine, and similar results have been obtained for coffee fermentation. Additionally, this study highlighted that temperature and fermentation time affect the flavor characteristics of coffee beverages [11]. This study aimed to explore the microbial diversity during anaerobic coffee fermentation.

Ochratoxin is a mycotoxin produced by various microorganisms in coffee bean. The threads and solutions for ochratoxin production have been discussed [19]. This study mainly explores the changes of bacterial species in the anaerobic coffee fermentation process under different temperature responses and evaluates the inhibitory potential of its metabolites on mycotoxin-producing bacteria, hoping to reduce the health risks of coffee drinkers through fermentation control.

## 2. Materials and Methods

### 2.1. Sample and Fermentation Process

All coffee beans used in this study were from a single batch. The Arabica coffee cherries (100 g) (control group) and those with 300 mL of 30% fructose (experimental group) were placed in a plastic bag and put in an anaerobic cylinder with an anaerobic pack. Different strains of microorganisms have different optimal temperatures for growth. For example, acetic acid bacteria and lactic acid bacteria can grow at higher temperatures, while fungi and yeasts grow better at lower temperatures. Therefore, the biosynthesis of metabolites is also important. It will change with the fermentation temperature, which will affect the coffee flavor [20]. This study used two different temperatures for coffee fermentation and explored its bacterial phase, organic acid metabolites, and the inhibition of mycotoxin species. The control group was fermented at 20 °C (C12 and C36), and the experimental group was fermented at 37 °C (E12 and E36), respectively. The fermented liquid from the bag was taken for full-length 16S rRNA sequencing analysis at 12 h and 36 h.

### 2.2. Full-Length 16S rRNA Analysis 

Total genomic DNA was extracted from the sample using the QIAamp PowerFecal DNA kit (Qiagen). PCR amplification was performed using the 16S primer F (5′-AGRGTTYGATYMTGGCTCAG-3′) and R (5′-RGYTACCTTGTTACGACTT-3′) and purified using AMPure PB Bead (PacBio, CA, USA). The SMRTbell adapter was attached to the purified PCR product and sequenced full-length 16S rRNA on the PacBio RS II SMRT DNA sequencing system (Pacific Biosciences, Menlo Park, CA, USA) using the P6-C4 chemistry. Repeat sequences were organized to generate Circular Consensus Sequencing (CCS) [21]. CCS data was analyzed using DADA2 to produce Amplicon Sequence Variants (ASVs). Species information was obtained by comparing the data with the NCBI database.

### 2.3. Inhibition of Coffee-Fermented Metabolites against Spore Germination of Ochratoxin-Produced Aspergillus niger

The ochratoxin-produced *Aspergillus niger* (BCRC33485) was purchased from Bioresource Collection and Research Center (BCRC, Food Industry Research and Development Institute, Hsin Chu, Taiwan). *A. niger* spores were isolated from 3-day-old cultured medium (including 55 mM glucose, 11 mM KH_2_PO_4_, 7 mM KCl, 178 nM H_3_BO_3_, 2 mM MgSO_4_, 76 nM ZnSO_4_, 70 mM NaNO_3_, 6.2 nM Na_2_MoO_4_, 18 nM FeSO_4_, 7.1 nM CoCl_2_, 6.4 nM CuSO_4_, 25 nM MnCl_2_, 0.5% yeast extract), and spore suspensions were filtered to remove hyphal fragments [22,23]. The inhibition of coffee fermented metabolites by C36h or E36h treatment against spore germination was evaluated. Briefly, 100 μL spore suspensions (10^4^/mL) was mixed with C36h- or E36h-fermented solution (100 μL) at 25 °C for 2 h. Subsequently, these mixed solutions were inoculated on potato dextrose agar (PDA) plates and incubated for 24 h.

### 2.4. Analysis of Organic Acids

Organic acids were measured using Dionex ICS-3000 ion exchange chromatographic system according to recent study mediated by AS1 autosampler and DC-2 Detector/Chromatography system equipped with an AMMS-ICE 300 suppressor (Thermo Fisher Scientific, Hvidovre, Denmark) [20]. The sample (10 μL) was separated on an IonPac ICE-AS6 IC ion exclusion column at 35 °C (250 mm × 9 mm, 8 μm particle size; Thermo Fisher Scientific, Hvidovre, Denmark) by an isocratic flow (0.2 mM HCl).

### 2.5. Statistical Analysis

The samples were triplicated, and results are presented as mean ± standard deviation. Non-parametric factorial Kruskal–Wallis (KW) sum-rank test, Graphical Phylogenetic Analysis (GraPhlAn), Non-metric Multidimensional Scaling (NMDS), Principal Component Analysis (PCA), Principal Co-ordinates Analysis (PCoA), Welch’s *t*-test and Spearman test were used to compare the relative abundance of the defining ASVs between groups. Statistical analysis and plotting were performed in R (3.4.0). *p* value ≤ 0.05 was considered significant [24].

## 3. Results and Discussion

### 3.1. Microbial Abundance and Diversity of Anaerobic Fermented Coffee

**Figure 1:**
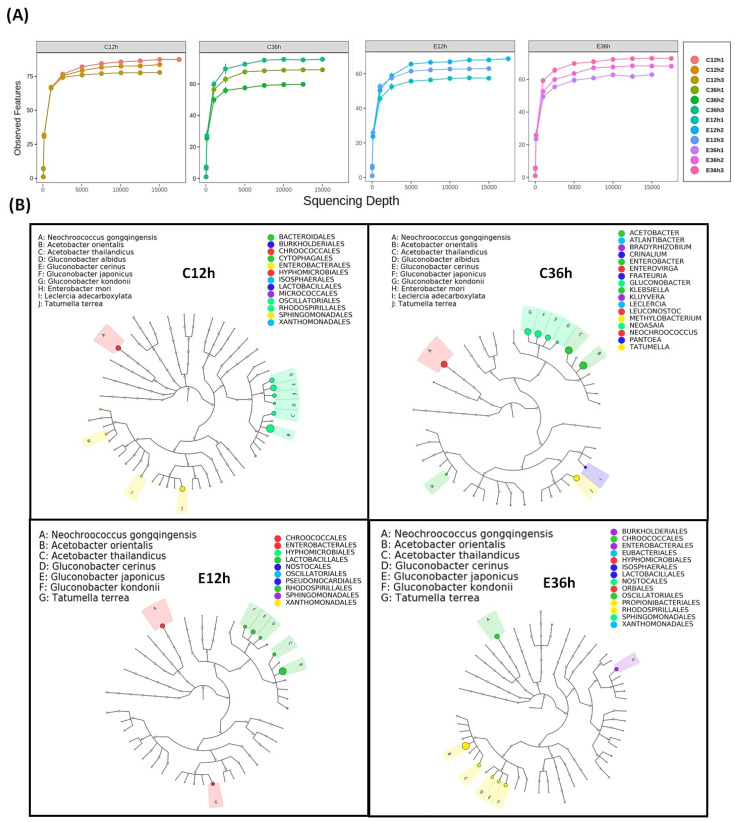
The microbial abundance of anaerobic fermented coffee. (**A**) Rarefaction analysis of ASVs. When the curve gradually becomes flat, it indicates that the amount of sequenced data is progressively reasonable. (**B**) Graphical Phylogenetic Analysis (GraPhlAn) of ASVs. The circles represent different taxonomic levels from inside to outside, and the size of circles resembles the species abundance. Different colors represent different phylum. Relatively high-abundance dominant species are indicated by solid circles. The fermentation was carried out for 12–36 h. The control group (n =3) was fermented at 20 °C (C12h and C36h), and the experimental group (n =3) was fermented at 37 °C (E12h and E36h).

As shown in Figure 1, the sample rarefaction analysis reveals the validity of the sequencing data, and when the curve tends to be smooth, the sequencing data becomes acceptable, indicating that sample detection is sufficient to study microbial diversity. The Graphical Phylogenetic Analysis (GraPhlAn) tool can highlight species evolutionary relationships and species richness [25]. The dominant species among the four fermentation groups is *Acetobacteraceae,* which includes *Acetobacter* and *Gluconobacter*. According to one study, *Acetobacter* and *Gluconobacter* are abundant in the first 12–20 h of the coffee fermentation process [26]. Additionally, *Enterobacteriaceae*, including *Enterobacter*, *Leclercia*, *Pantoea*, and *Tatumella,* have been mentioned in anaerobic fermentation processes [27], which may be because *Enterobacteriaceae* decompose sugars and monosaccharides through the production of many degradable enzymes under anaerobic fermentation conditions. Additionally, they produce enzymes involved in amino acid degradation [28], and reducing sugars and amino acids are important precursors of the Maillard reactions [29].

**Figure 2:**
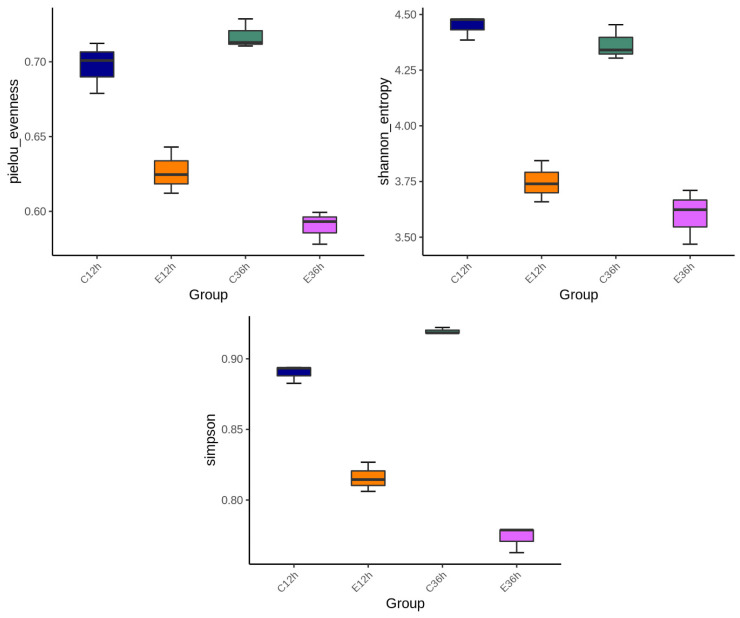
The microbial abundance and diversity of anaerobic fermented coffee. Alpha diversity including Pielou, Shannon and Simpsons indices were used to evaluate microbial abundance and alpha-diversity within groups. The fermentation was carried out for 12–36 h. The control group was fermented at 20 °C (C12h and C36h), and the experimental group was fermented at 37 °C (E12h and E36h), respectively.

**Figure 3:**
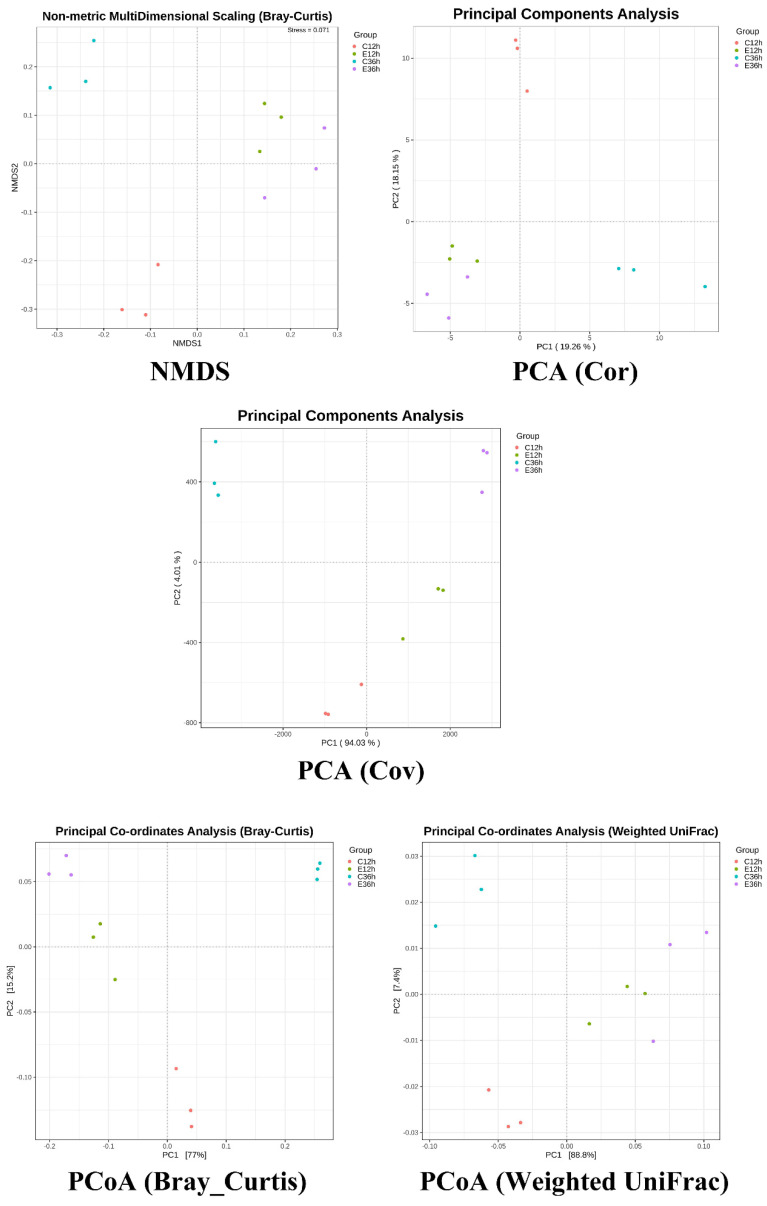
The microbial abundance and beta-diversity of anaerobic fermented coffee. The fermentation was carried out for 12–36 h. The control group was fermented at 20 °C (C12h and C36h), and the experimental group was fermented at 37 °C (E12h and E36h), respectively.

Alpha diversity, including the Pielou, Shannon, and Simpson indices, with higher index values indicates higher microbial diversity. At 12 h of fermentation, anaerobic fermentation at 20 °C (C12) exhibited more diversity than that at 37 °C (E12) (Figure 2). For the 36 h fermentation groups, the difference in alpha diversity at different temperatures became more significant. The trends in diversity at different temperatures shifted as fermentation time increased, with the indices at lower temperatures increasing, whereas the indices at higher temperatures gradually decreased. In this study, the alpha diversity indices revealed that fermentation at 20 °C (C36) was higher than that at 37 °C (E36). This finding is consistent with previous studies [11,14]. This phenomenon may be due to the slower change in microbial composition at lower initial fermentation temperatures, and some studies have observed that diversity presents a hump pattern over time [4]. Higher fermentation temperatures may result in selective conditions for certain bacteria, leading to the death of some bacteria and formation of dominant bacteria, causing a decrease in diversity [11,14,30,31]. Figure 3 showed the beta-diversity results. Irrespective of whether it is the NMDS of a non-linear model, PCA of a linear model, PCoA calculated from the distance matrix (Bray_Curtis), or PCoA that accounts for the weighted proportions of each species (Weighted UniFrac), fermentation at 37 °C in E12 and E36 group were more similar, whereas the fermentation at 20 °C had a greater distance, indicating that the fermentation time at 20 °C had a greater impact on the microbial community.

### 3.2. Variations in the Structure of Microbial Communities in Anaerobic Fermented Coffee

**Figure 4:**
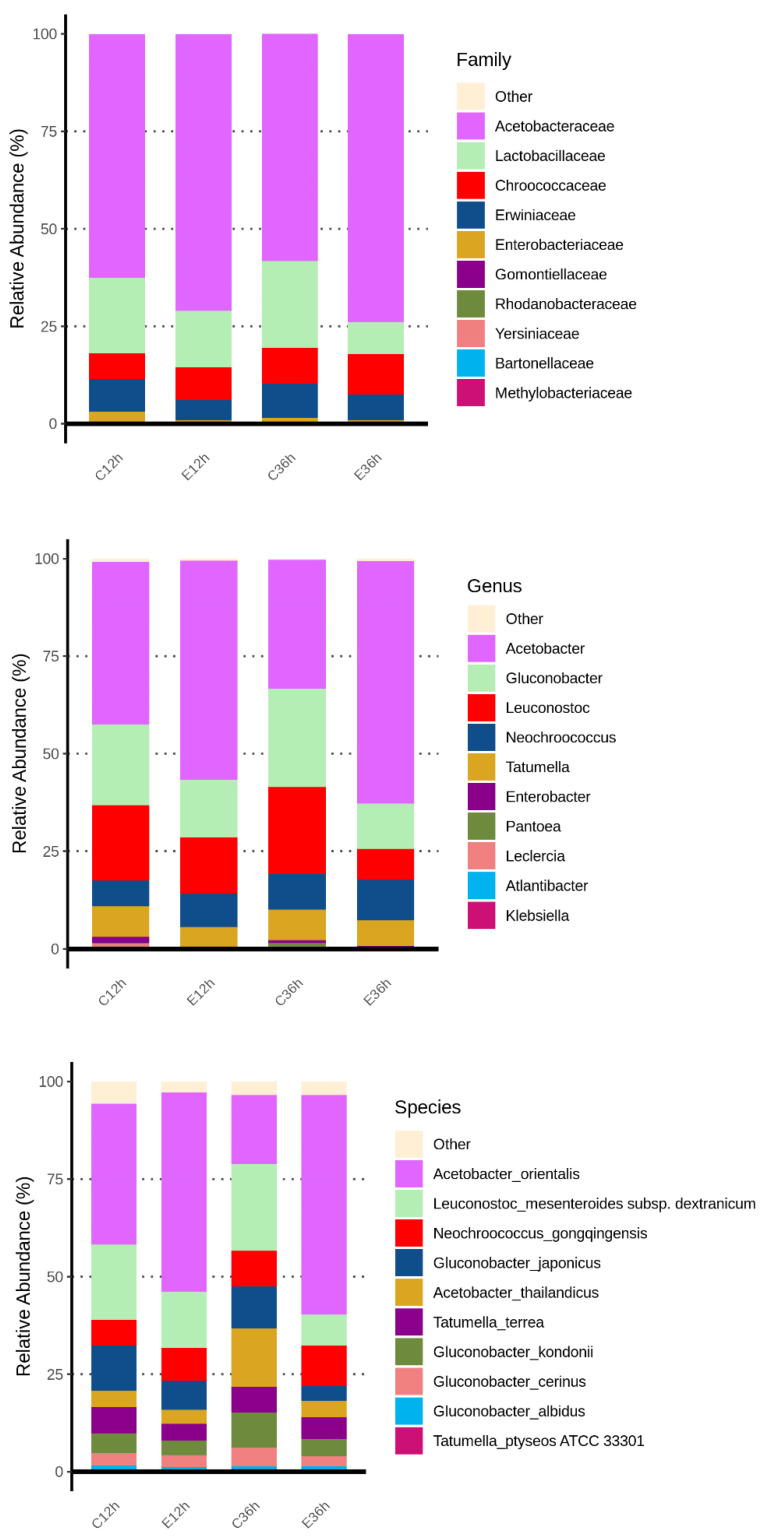
Relative abundance (%) bar chart of bacterial at family, genus, and species in anaerobic fermented coffee. Top 10 classifications for each level for each group are shown. Taxa not in the top 10 are grouped together in the ‘others’ category.

The top ten family, genus, and species in abundance during the anaerobic fermentation process were Acetobacteraceae, Lactobacillaceae, and Enterobacteriaceae for the family; *Acetobacter*, *Gluconobacter*, and *Leuconostoc* for the genera; and the main bacteria were acetic acid bacteria and lactic acid bacteria (Figure 4). This result was similar to that reported by Pereira et al. [32]. Moreover, De Melo Pereira et al. mentioned that *Leuconostoc* is a commonly occurring genus in coffee fermentation worldwide, and that lactic acid bacteria assisted with the breakdown of the mucilage layer [33], and a lot of *Leuconostoc* was found in coffee cherry in Taiwan [13]. Acetobacteraceae are widely present in various fermented foods [34], and acetic acid bacteria are commonly found in dry processes [35]. This is because of acetic acid bacteria are abundant in coffee cherry peels, and this fermentation process ferments coffee cherries with their peels, making them the dominant microorganisms. However, the effects of *Acetobacter* and *Gluconobacter* species on coffee fermentation under anaerobic conditions remain unclear [36]. Moreover, from the relative abundance bar chart, it can be seen that Acetobacter had a better growth rate at higher temperatures, whereas *Leuconostoc* grew better at lower temperatures. It is because the growth of *Acetobacter* was limited at low temperatures and gradually lost its advantages during fermentation. However, *Leuconostoc*, which is commonly found in dairy and meat fermentation, had good low-temperature tolerance and could grow slowly even at low temperatures, gradually increasing during low-temperature fermentation [37,38]. The *Enterobacter* was commonly found as a dominant genus in the early to mid-stages of anaerobic fermentation [27,28]. The *Klebsiella*, *Gluconobacter*, *Enterobacter*, *Tatumella*, *Pantoea*, and *Serratia* have also been found in other anaerobic fermentation processes, and these genera mostly originate from water, soil, plants, and insects [5,32,39,40,41].

**Figure 5:**
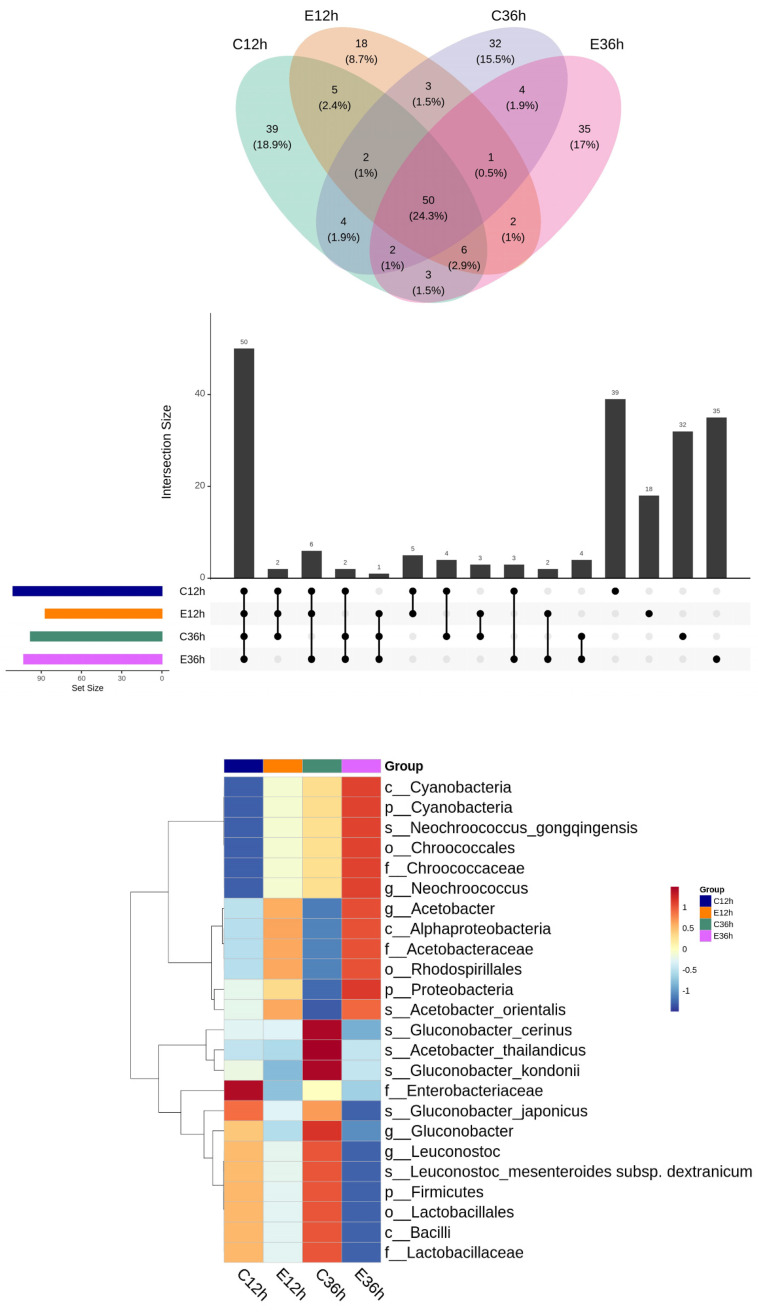
Microbial relative relationship among groups of anaerobic fermented coffee. Venn diagram and UpSet plot (**top**) and heatmap (**bottom**).

A Venn diagram and an UpSet plot were used to examine the amount and species of common and distinctive ASVs in various groups of anaerobic fermented coffee. The lower section of the plot may be used to determine the group being investigated as a consequence of the UpSet plot. Combining the UpSet plot with the Venn diagram revealed that there were 50 common species in all groups, and 39 species were specific to C12h, 18 to E12h, 32 to C36h, and 35 to E36h (Figure 5).

### 3.3. Statistical Analysis of Microbial Communities in Anaerobic Fermented Coffee

**Figure 6:**
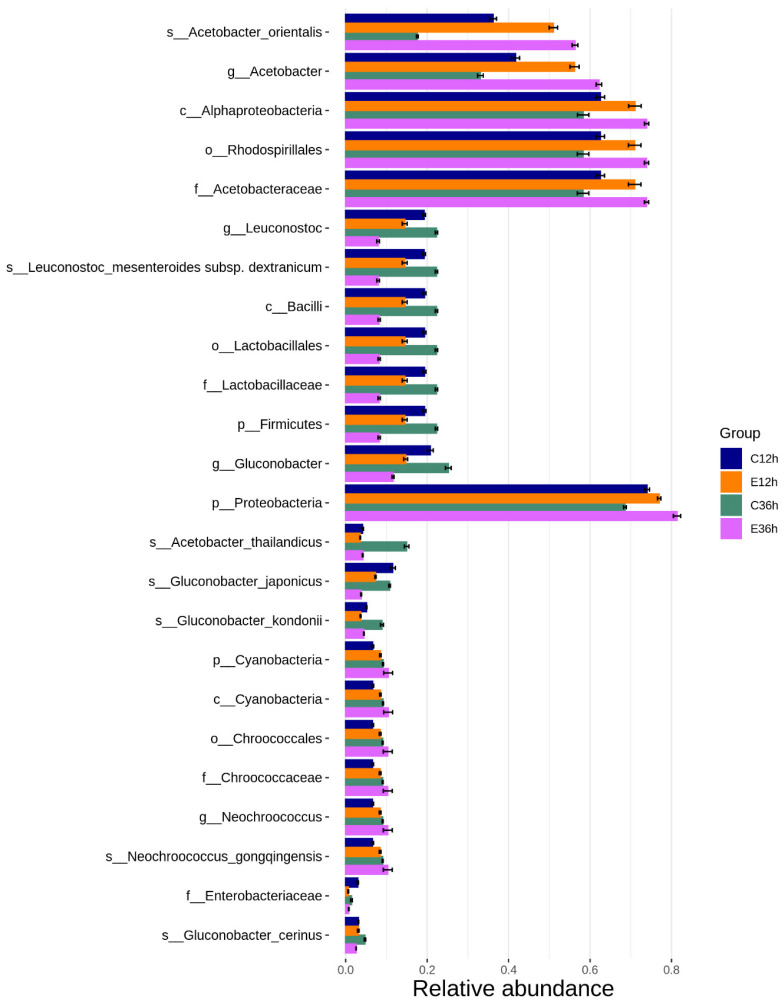
Biomarkers of anaerobic fermented coffee. Linear discriminant analysis Effect Size (LEfSe) was used to estimate the impact of each species abundance on the difference effect and find the communities or species that have significant differences in the sample division. The Linear discriminant analysis (LDA) distribution histogram shows the species as biomarkers whose LDA Score is greater than the threshold value (default: 4) with statistical differences between groups. The length of the histogram represents the impact of different species. The relative abundance of biomarkers was drawn as a clustering heatmap of groups. The abundance of groups is the average abundance of all samples in the groups.

Linear discriminant analysis effect size (LEfSe) was used to identify microbial species with significant differences in abundance. A non-parametric factorial Kruskal–Wallis sum-rank test was used to identify significant differences in abundance, and linear discriminant analysis (LDA) was used to estimate the effect size of each species on the difference. LEfSe helps identify communities or species that have a significant impact on sample categorization [42]. Species with LDA scores greater than the threshold were considered biomarkers, and there were statistical differences among the groups. The figure on the right shows a heat map of the biomarkers plotted according to their relative abundance (Figure 6). Clustering results are shown on the left side of the heatmap and divided into the following four main groups from top to bottom: *Neochroococcus*, *Acetobacter*, *Gluconobacter*, and *Leuconostoc*. The *Neochroococcus* increased with time in both the temperature groups, suggesting that it may be a plant or water contaminant. *Acetobacter* decreased over time in the low-temperature fermentation group but gradually increased in the high-temperature fermentation group. The *Gluconobacter* and *Leuconostoc* increased gradually in the low-temperature group but decreased gradually in the high-temperature group. The *Leuconostoc* is a common dominant bacterial species in the later stages of fermentation [28]. This phenomenon was also observed in this study. There is a significant association with Acetobacter at 37 °C fermentation, which may be related to its generally better heat resistance [43].

**Figure 7:**
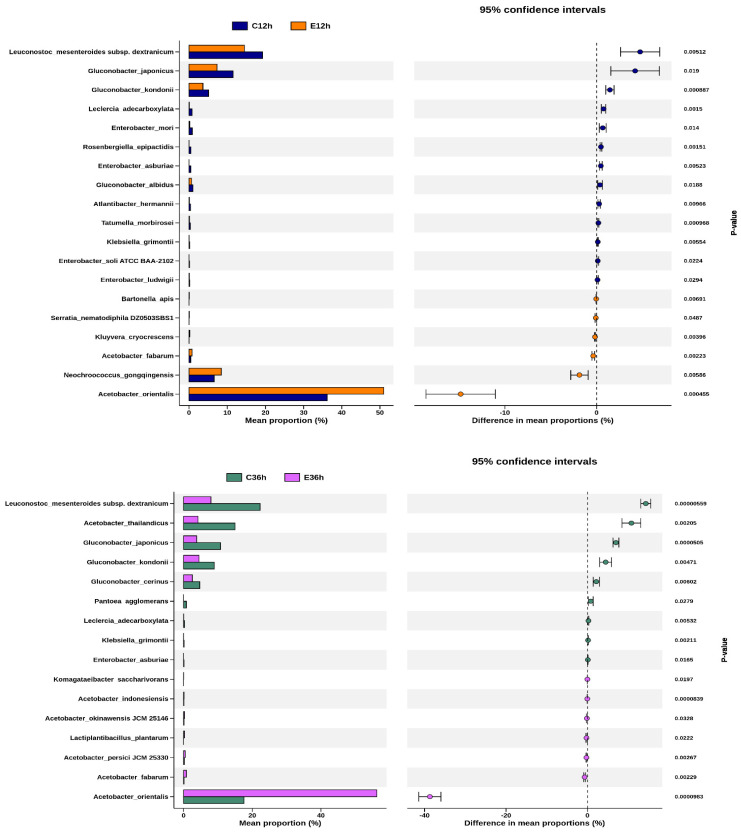

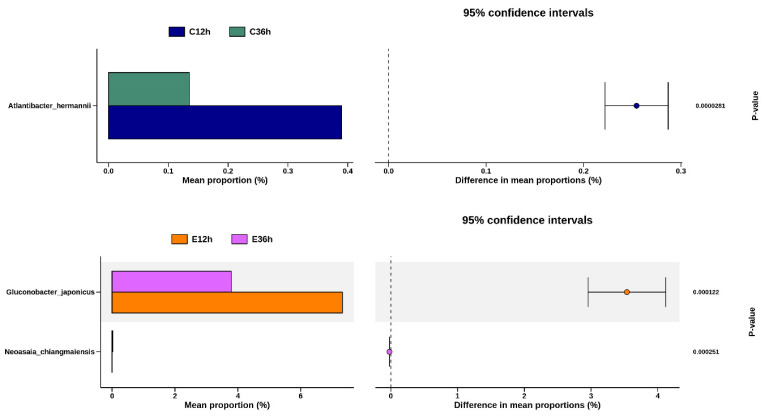
Differential species between any two groups of anaerobic fermented coffee. Welch’s *t*-test between C12h and E12h, and between C36h and E36h. Welch’s *t*-test between C12h and C36h, and between E12h and E36h. The left chart represents the mean abundance ratio of different significantly species in the two groups. The right chart represents the confidence of the difference between groups. The leftmost endpoint of each circle in the graph represents the lower bound of the 95% confidence interval for the mean difference, and the rightmost endpoint represents the upper bound. The center of the circle represents the difference between the mean values. The group with high mean value would be represented by the circle color. The value on the right is the *p*-value of the significance test between groups of different species, and *p*-value < 0.05 indicates that the difference is significant.

Welch’s *t*-test allowed us to identify the species with the greatest differences between the groups. A comparison is shown between the same fermentation time but different fermentation temperatures (Figure 7). The species with significant and representative differences were Leuconostoc, Gluconobacter, and Acetobacter. After 12 h of fermentation, Leuconostoc and Gluconobacter were higher in low-temperature fermentation, whereas Acetobacter was higher in high-temperature fermentation. After 36 h of fermentation, the proportion difference between these species became even more significant. The species with the most significant changes with increasing fermentation time at the same temperature were, at low temperatures, Atlantibacter hermannii of Enterobacteriaceae, and at high temperatures, Gluconobacter japonicas of Acetobacteraceae, both of which showed a significant decrease.

**Figure 8:**
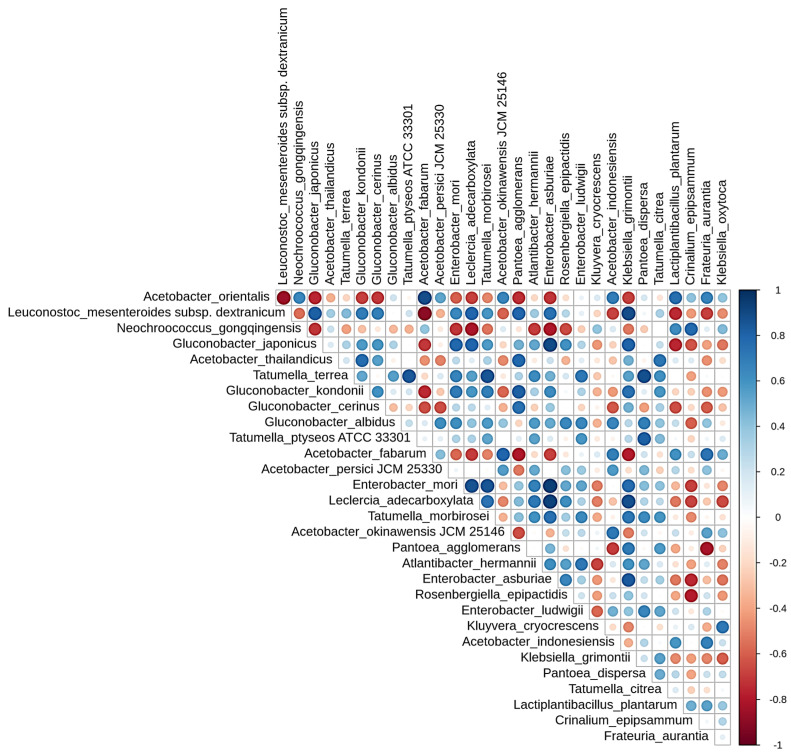
Spearman’s correlation matrix of the abundant bacterial in anaerobic fermented coffee. Blue represents positive correlation and red represents negative correlation. The beginning letters represents that the species are respectively annotated at the level of kingdom, phylum, class, order, family and, genus.

Spearman’s correlation matrix (Figure 8) was used to calculate and examine the correlation coefficients among the top 30 species in the ASVs species abundance table. The plot reveals a dependency or antagonistic relationship between the dominant species [14]. By observing the biomarkers marked, Neochroococcus was found negatively correlated with the other genera (Figure 8). Additionally, Acetobacter showed a negative correlation with Gluconobacter and Leuconostoc, but Gluconobacter was positively correlated with Leuconostoc, resulting in lactic acid being oxidized to form acetic acid mediated by Acetobacter [44]. In summary, lower fermentation temperatures have a higher correlation with microorganisms, such as Leuconostoc and Gluconobacter. Lactic acid bacteria are essential components of contemporary specialty coffee processing [33]. These bacteria metabolize sugars in the mucilage layer to produce acids, thereby reducing the pH and causing the breakdown of pectins [45]. This metabolic action effectively shortens the drying process after harvesting [46]. Different acids, alcohols, esters, aldehydes, and ketones produced by the metabolism of lactic acid bacteria also serve as aroma compounds and precursors to the final flavor of coffee drinks, contributing to floral, fruit, and cream-like aromas [47]. The results of this study confirm that low-temperature, long-term anaerobic fermentation promotes the growth of lactic acid bacteria. Although the contribution of Acetobacter to coffee flavor associated with higher fermentation temperatures remains unclear [36], its suitable growth temperature differs from that of lactic acid bacteria, which can lead to a different coffee flavor profile.

### 3.4. Inhibition of Coffee-Fermented Metabolites against Spore Germination of Ochratoxin-Produced Aspergillus niger

**Figure 9:**
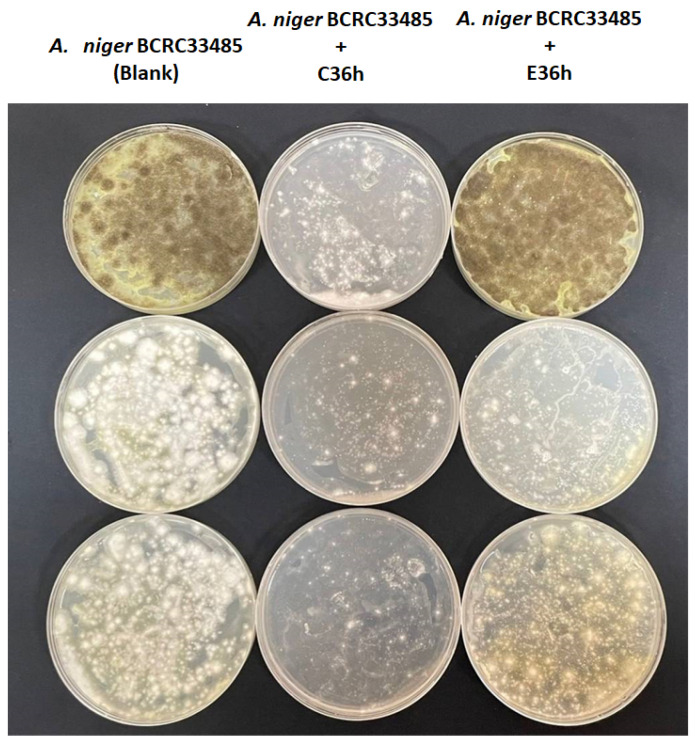
Inhibition of coffee-fermented metabolites against spore germination of ochratoxin-produced *Aspergillus niger* (n = 3).

Mycotoxins are harmful substances, and ochratoxin is a mycotoxin produced by Aspergillus spp. and which was always found in coffee. The preventive strategies of ochratoxin production are inhibiting spore germination and fungal growth for ochratoxin production [48]. *A. niger* is one of the Aspergillus spp. frequently found in coffee and known to produce ochratoxin. This project uses ochratoxin-producing bacteria (*Aspergillus niger* BCRC33485) as the target control object and explores the changes in bacterial species under fermentation at different temperatures and their inhibitory effects on ochratoxin-produced microorganisms. As shown in Figure 9, the growth of *A. niger* was markedly suppressed after C36h treatment. Recently, the several microbes have been found to inhibit ochratoxin-producing-Aspergillus spp. growth and reduce ochratoxin level, including Candida friedrichii, Candida intermedia, and Lachancea thermotolerans [49]. Moreover, lactic acid bacteria (Lactobacillus acidophilus and Lactobacillus rhamnosus) have also been reported to decrease ochratoxin production [50]. 

On the other hand, acids in coffee are generally divided into two categories including organic acids and chlorogenic acids. Various organic acids identified from coffee have been reported, and a study quantified citric acid, malic acid, and quinic acid as prominent in coffee [51]. In addition to increasing the sour taste, different organic acids also have different organoleptic properties. For example, citric acid, acetic acid, formic acid, malic acid, quinine, pyrrolic acid, succinic acid, fumaric acid, tartaric acid, and lactic acid can not only present different sour taste characteristics but also present special aroma quality. In addition, there are also reports that acetic acid can present vinegar aroma, pyruvate, caramel flavor, formic acid, in addition to pungent sour taste, and also has fermentation aroma and bitter taste [52,53]. The various organic acids (including acetic acid, citric acid, formic acid, lactic acid, and malic acid) in anaerobic fermented coffee were investigated as shown in Table 1. We found that acetic acid was a major organic acid both in C36h and E36h groups, and the level of lactic acid was abundant in the C36h group. Gluconobacter produces acetic acid like acetic acid bacteria, but Acetobacter produces acetic acid in an environment containing alcohols, and Gluconobacter can decompose carbohydrates to produce acetic acid. In this study, we found that the number of Gluconobacter and Leuconostoc was significantly present in the C36h group, but the microorganisms in the E36h group were mainly Acetobacter as the main fermentation flora (Figure 4), indicating that fermented at 20 °C (C36h) and at 37 °C (E36h) regulated different bacterial genus growth and resulted in different metabolites production.

The use of organic acids in food or fermentation can be considered an alternative that acts as a preservative to limit mold growth in food and reduce the potential for mycotoxin contamination. Susceptibility of Aspergillus spp. to acetic and sorbic acids has been reported, and the potential of sorbic acid reduced ochratoxin A production could be developed [54]. Recently, ochratoxin A production was potentially inhibited by bio-control strategy such as fermentation [55], and several studies reported that lactic acid bacteria (Leuconostoc mesenteroides) could suppress ochratoxigenic fungi [56,57].

## 4. Conclusions

In this study, microbial diversity during the anaerobic fermentation of coffee at different temperatures and times was investigated. Full-length 16S rRNA analysis showed that the dominant microbial community, formed at higher fermentation temperatures, was *Acetobacter* of the acetic acid bacteria group, whereas the dominant microbial community, at lower temperatures, was mainly *Leuconostoc* of the lactic acid bacteria group and *Gluconobacter* of the acetic acid bacteria group. Additionally, changes in the microbial community were slow at low temperatures and were associated with the temperature tolerance of each species. In conclusion, different fermentation temperatures can lead to different microbial communities during the anaerobic fermentation of coffee, and the results of this study can be used as a basis for the development of future coffee fermentation processes.

## Figures and Tables

**Table 1:** The organic acids in anaerobic fermented coffee at 20 °C (C36h) and at 37 °C (E36h) treatment.

Groups	C36h			E36h		
	mg/mL					
Acetic acid	0.257	±	0.034 ^b^	0.418	±	0.012 ^a^
Citric acid	0.141	±	0.068	0.183	±	0.025
Formic acid	0.107	±	0.026 ^a^	0.067	±	0.011 ^b^
Lactic acid	0.279	±	0.031 ^a^	0.107	±	0.008 ^b^
Malic acid	0.162	±	0.019	0.131	±	0.006

Data were shown as mean ± SD (n = 3). Significant difference was indicated by different letters (*p* < 0.05).

## Data Availability

Data is contained within the article.
